# Supplementary material for: Inflammatory Markers as Predictors of Shunt Dependency and Functional Outcome in Patients with Aneurysmal Subarachnoid Hemorrhage
Source: Biomedicines. 2023 Mar 23;11(4):997. doi: 10.3390/biomedicines11040997 (PMC10135965; doi:10.3390/biomedicines11040997)
Supplement: Supplementary file 1 [file biomedicines-11-00997-s001.zip › Rostgaard et al, SAH_SUPPL file1.pdf]

Supplemental file S1, Table S1
Predictors of shunt dependency (not reaching lower confidence limit of ≥0.7)

Name	Time	EVD weaned - mean (SD) [n]	VP-shunt - mean (SD) [n]	AUC (95%CI)	Cut-off	Sensitivity	Specificity
IL8	Start	12.56 (1.04) [19]	10.44 (1.58) [12]	0.86 (0.69-1)	11.06	0.75	0.89
	End	12.4 (1.37) [19]	11.68 (1.24) [12]	0.66 (0.46-0.86)	12.22	0.75	0.63
	Change	0.01 (0.09) [19]	-0.06 (0.09) [12]	0.74 (0.55-0.93)	-0.02	0.83	0.58
VEGFA	Start	9.96 (0.83) [19]	10.23 (1.11) [12]	0.56 (0.34-0.77)	11.69	0.17	1.00
	End	9.38 (1.03) [19]	10.34 (0.83) [12]	0.83 (0.68-0.98)	9.50	1.00	0.68
	Change	0.04 (0.08) [19]	0 (0.08) [12]	0.68 (0.49-0.88)	0.04	0.83	0.63
CD8A	Start	5.37 (1.1) [19]	5.11 (1.39) [12]	0.61 (0.4-0.83)	5.18	0.75	0.58
	End	4.98 (1.02) [19]	5.89 (0.54) [12]	0.76 (0.59-0.93)	5.25	1.00	0.58
	Change	0.02 (0.07) [19]	-0.04 (0.08) [12]	0.73 (0.53-0.93)	-0.03	0.67	0.84
MCP 3	Start	5.76 (1.59) [17]	5.16 (1.91) [10]	0.68 (0.43-0.93)	5.26	0.80	0.71
	End	5.54 (1.89) [18]	4.72 (1.45) [11]	0.62 (0.41-0.83)	5.55	0.82	0.44
	Change	0.01 (0.15) [17]	0.03 (0.12) [9]	0.52 (0.28-0.77)	0.00	0.67	0.47
CDCP1	Start	3.75 (0.78) [19]	3.56 (0.58) [12]	0.56 (0.34-0.78)	3.53	0.50	0.68
	End	3.33 (0.61) [19]	3.79 (0.57) [12]	0.68 (0.48-0.87)	3.58	0.75	0.68
	Change	0.02 (0.06) [19]	-0.01 (0.03) [12]	0.71 (0.53-0.9)	0.02	0.92	0.53
OPG	Start	10.31 (1.31) [19]	9.79 (1.26) [12]	0.61 (0.4-0.82)	9.77	0.58	0.68
	Change	0.07 (0.1) [19]	-0.05 (0.09) [12]	0.84 (0.7-0.98)	0.01	0.83	0.74
LAP TGF beta 1	Start	4.39 (0.82) [19]	4.24 (0.92) [12]	0.6 (0.38-0.82)	4.36	0.75	0.58
	Change	0.03 (0.07) [19]	-0.02 (0.06) [12]	0.77 (0.59-0.96)	-0.01	0.67	0.89
uPA	Start	7.91 (0.83) [19]	7.86 (0.74) [12]	0.52 (0.3-0.73)	7.52	0.50	0.68
	End	8.34 (1.13) [19]	9.47 (1.1) [12]	0.74 (0.56-0.91)	8.67	0.83	0.58
	Change	-0.04 (0.11) [19]	-0.08 (0.07) [12]	0.74 (0.56-0.93)	-0.04	0.92	0.63

IL6	Start	9.75 (1.72) [19]	8.51 (1.99) [12]	0.69 (0.48-0.91)	9.29	0.83	0.74
	End	8.54 (2.13) [19]	8.44 (1.84) [12]	0.54 (0.33-0.75)	7.94	0.58	0.63
	Change	0.06 (0.14) [19]	0.01 (0.13) [12]	0.68 (0.47-0.89)	0.07	0.75	0.68
MCP 1	Start	14.69 (0.51) [19]	14.79 (0.44) [12]	0.6 (0.37-0.83)	14.88	0.67	0.63
	End	14.4 (0.91) [19]	14.83 (0.32) [12]	0.61 (0.41-0.81)	14.47	0.92	0.42
	Change	0.02 (0.05) [19]	0 (0.03) [12]	0.64 (0.43-0.84)	0.02	0.83	0.53
CXCL11	Start	4.64 (0.9) [19]	4.74 (1) [12]	0.5 (0.28-0.73)	4.42	0.58	0.63
	End	5.58 (1.23) [19]	6.17 (0.59) [12]	0.68 (0.48-0.87)	5.77	0.75	0.68
	Change	-0.08 (0.13) [19]	-0.07 (0.07) [12]	0.52 (0.31-0.73)	-0.07	0.67	0.53
TRAIL	Start	3.15 (1.1) [17]	3.26 (0.92) [12]	0.57 (0.35-0.79)	3.21	0.58	0.65
	End	2.21 (0.32) [11]	2.23 (0.4) [12]	0.5 (0.25-0.75)	1.83	1.00	0.18
	Change	0.04 (0.07) [11]	0.05 (0.06) [12]	0.52 (0.27-0.76)	0.03	0.58	0.55
CXCL9	Start	3.95 (0.85) [19]	4.09 (0.82) [12]	0.54 (0.32-0.76)	2.96	1.00	0.21
	End	5.95 (1.43) [19]	7.04 (1.11) [12]	0.72 (0.53-0.91)	6.66	0.75	0.68
	Change	-0.15 (0.14) [19]	-0.15 (0.07) [12]	0.55 (0.34-0.77)	-0.10	0.92	0.32
CST5	Start	5.28 (0.27) [19]	5.32 (0.22) [12]	0.51 (0.29-0.73)	4.91	1.00	0.16
	Change	0 (0.02) [19]	-0.01 (0.01) [12]	0.74 (0.54-0.94)	-0.01	0.75	0.84
OSM	Start	6.35 (1.78) [19]	6.36 (1.89) [12]	0.54 (0.32-0.77)	5.43	0.50	0.79
	End	5.47 (1.46) [19]	5.35 (0.98) [12]	0.57 (0.36-0.78)	6.92	1.00	0.26
	Change	0.04 (0.14) [19]	0.05 (0.1) [12]	0.52 (0.31-0.74)	0.03	0.58	0.63
CXCL1	Start	10.07 (1.12) [19]	8.21 (1.59) [12]	0.83 (0.63-1)	8.18	0.75	1.00
	End	10.76 (1.76) [19]	10.75 (1.34) [12]	0.52 (0.31-0.73)	10.94	0.58	0.63
	Change	-0.05 (0.08) [19]	-0.13 (0.09) [12]	0.75 (0.57-0.92)	-0.06	0.75	0.68
CCL4	Start	7.93 (2.57) [19]	8.32 (1.88) [12]	0.56 (0.35-0.77)	8.17	0.75	0.58
	End	7.35 (1.83) [19]	6.42 (0.69) [12]	0.64 (0.43-0.84)	7.10	0.92	0.53

	Change	0.03 (0.19) [19]	0.09 (0.09) [12]	0.59 (0.38-0.79)	0.04	0.83	0.53
SCF	Start	4.54 (0.87) [19]	4.83 (0.8) [12]	0.63 (0.42-0.83)	4.26	0.83	0.58
	Change	0.02 (0.06) [19]	-0.02 (0.06) [12]	0.73 (0.54-0.92)	0.02	0.92	0.53
IL18	Start	4.45 (1.52) [19]	4.44 (1.16) [12]	0.5 (0.29-0.72)	6.44	1.00	0.16
	End	5 (1.4) [19]	5.04 (0.77) [12]	0.52 (0.31-0.73)	5.82	0.83	0.42
	Change	-0.06 (0.17) [19]	-0.03 (0.07) [12]	0.53 (0.31-0.74)	-0.04	0.67	0.53
TGF alpha	Start	5.44 (0.83) [19]	5.9 (0.97) [12]	0.65 (0.44-0.86)	5.11	0.83	0.53
	End	4.65 (0.5) [19]	5.25 (0.4) [12]	0.83 (0.69-0.97)	4.61	1.00	0.53
	Change	0.05 (0.06) [19]	0.04 (0.06) [12]	0.56 (0.34-0.79)	-0.02	0.25	1.00
MCP 4	Start	9.82 (1.74) [19]	10 (1.23) [12]	0.55 (0.34-0.76)	10.28	0.50	0.74
	End	10.87 (1.93) [19]	11.61 (1.1) [12]	0.67 (0.47-0.86)	10.98	0.83	0.58
	Change	-0.09 (0.15) [19]	-0.08 (0.09) [12]	0.51 (0.3-0.72)	0.02	0.92	0.26
CCL11	Start	4.29 (1.13) [18]	4.65 (0.79) [12]	0.62 (0.41-0.82)	3.73	0.92	0.39
	End	3.07 (0.51) [19]	3.33 (0.25) [12]	0.72 (0.53-0.91)	3.09	0.92	0.68
	Change	0.08 (0.06) [18]	0.07 (0.05) [12]	0.53 (0.32-0.75)	0.16	1.00	0.17
FGF 5	Start	2.69 (0.92) [14]	2.32 (0.61) [7]	0.67 (0.35-0.99)	1.95	0.57	1.00
	End	2.71 (0.65) [14]	2.82 (0.53) [12]	0.62 (0.38-0.86)	2.71	0.75	0.71
	Change	0 (0.06) [13]	-0.03 (0.03) [7]	0.6 (0.35-0.86)	0.02	1.00	0.38
MMP 1	Start	11.37 (2.27) [19]	12.06 (1.81) [12]	0.59 (0.38-0.8)	10.32	0.92	0.32
	End	10.38 (2.48) [19]	10.68 (1.72) [12]	0.54 (0.32-0.75)	8.43	1.00	0.21
	Change	0.08 (0.18) [19]	0.06 (0.11) [12]	0.5 (0.29-0.71)	0.03	0.75	0.53
LIF R	Start	3.08 (0.5) [19]	3.05 (0.37) [12]	0.52 (0.29-0.74)	3.25	0.42	0.79
	End	2.78 (0.45) [19]	3.07 (0.4) [12]	0.67 (0.48-0.87)	2.60	0.92	0.42
	Change	0.02 (0.03) [19]	0 (0.02) [12]	0.74 (0.55-0.92)	0.02	0.83	0.63
	Start	9.5 (1.26) [19]	9.58 (0.98) [12]	0.52 (0.31-0.73)	8.77	0.83	0.32

CCL19	End	11.01 (1.45) [19]	12.2 (0.36) [12]	0.76 (0.59-0.93)	11.65	1.00	0.53
	Change	-0.12 (0.15) [19]	-0.13 (0.07) [12]	0.61 (0.41-0.82)	-0.08	0.92	0.53
IL 10RB	Start	2.64 (0.52) [16]	2.58 (0.51) [10]	0.52 (0.28-0.77)	2.49	0.60	0.62
	End	2.72 (0.48) [17]	3.34 (0.46) [12]	0.82 (0.67-0.98)	3.15	0.75	0.82
	Change	-0.01 (0.05) [16]	-0.04 (0.04) [10]	0.77 (0.57-0.97)	-0.02	0.90	0.69
IL 18R1	Start	4.75 (1.2) [18]	4.5 (1.04) [12]	0.57 (0.36-0.79)	4.39	0.67	0.56
	End	3.65 (0.71) [16]	4.01 (0.5) [12]	0.71 (0.5-0.92)	3.43	1.00	0.56
	Change	0.06 (0.11) [16]	0.02 (0.07) [12]	0.71 (0.5-0.92)	0.03	0.75	0.81
PD L1	Start	3.92 (0.66) [15]	3.93 (0.5) [6]	0.57 (0.25-0.89)	4.12	0.50	0.87
	End	3.8 (0.47) [16]	4.03 (0.36) [12]	0.7 (0.5-0.91)	3.82	0.75	0.75
	Change	0 (0.05) [14]	0 (0.04) [6]	0.52 (0.21-0.84)	0.00	0.67	0.57
CXCL5	Start	9.89 (1.57) [19]	8.93 (2.17) [12]	0.69 (0.47-0.91)	9.47	0.75	0.74
	End	9.67 (2.27) [19]	9.03 (1.47) [12]	0.6 (0.4-0.8)	11.67	1.00	0.26
	Change	0.03 (0.16) [19]	0 (0.12) [12]	0.59 (0.38-0.8)	0.10	0.83	0.37
TRANCE	Start	1.69 (0.63) [8]	1.5 (0.46) [6]	0.6 (0.28-0.93)	1.43	0.67	0.62
HGF	Start	8.02 (0.62) [19]	7.98 (0.86) [12]	0.57 (0.34-0.81)	7.81	0.67	0.74
	End	7.81 (0.88) [19]	8.78 (0.74) [12]	0.78 (0.61-0.95)	8.82	0.50	0.95
	Change	0.01 (0.06) [19]	-0.04 (0.06) [12]	0.73 (0.55-0.91)	-0.02	0.75	0.68
IL 12B	Start	2.01 (0.88) [19]	2.2 (0.86) [12]	0.56 (0.35-0.78)	1.76	0.67	0.53
	End	2.86 (1.25) [19]	3.15 (0.81) [12]	0.59 (0.39-0.8)	2.17	1.00	0.32
	Change	-0.05 (0.1) [19]	-0.05 (0.06) [12]	0.52 (0.31-0.73)	-0.13	1.00	0.26
MMP 10	Start	7.08 (1.49) [19]	6.41 (1.97) [12]	0.66 (0.44-0.89)	5.54	0.50	0.89
	End	5.8 (1.31) [19]	6.74 (0.86) [12]	0.73 (0.55-0.91)	5.71	1.00	0.47
	Change	0.11 (0.16) [19]	-0.01 (0.1) [12]	0.8 (0.63-0.98)	0.01	0.75	0.84
IL10	Start	4.32 (1.19) [17]	3.87 (1.07) [11]	0.63 (0.39-0.86)	3.24	0.55	0.88

	End	3.16 (0.32) [8]	3.14 (0.49) [7]	0.55 (0.22-0.88)	2.88	0.43	0.88
TNF	Start	1.91 (1.05) [18]	1.54 (0.68) [11]	0.58 (0.36-0.79)	3.09	1.00	0.22
	End	3.02 (1.99) [19]	1.84 (0.34) [12]	0.6 (0.4-0.81)	2.36	1.00	0.42
	Change	-0.08 (0.14) [18]	-0.02 (0.04) [11]	0.65 (0.44-0.85)	-0.03	0.73	0.61
CCL23	Start	6.41 (0.81) [19]	5.93 (0.92) [12]	0.66 (0.45-0.87)	6.02	0.58	0.74
	End	7.28 (1.46) [19]	8.14 (1.09) [12]	0.68 (0.49-0.87)	7.01	0.92	0.47
	Change	-0.07 (0.09) [19]	-0.11 (0.09) [12]	0.68 (0.48-0.87)	-0.05	0.92	0.53
CD5	Start	3.56 (1.43) [19]	2.76 (1.29) [12]	0.71 (0.51-0.9)	2.74	0.75	0.68
	End	3.26 (1.13) [19]	3.07 (0.62) [12]	0.54 (0.33-0.75)	3.44	0.92	0.37
	Change	0 (0.13) [19]	-0.01 (0.09) [12]	0.68 (0.47-0.89)	-0.01	0.75	0.79
CCL3	Start	7.7 (2.47) [19]	7.47 (1.78) [12]	0.5 (0.29-0.71)	7.01	0.67	0.53
	End	8.94 (2.76) [19]	6.92 (0.67) [12]	0.68 (0.49-0.88)	7.61	0.92	0.58
	Change	-0.09 (0.19) [19]	0.02 (0.09) [12]	0.74 (0.56-0.91)	-0.03	0.83	0.68
Flt3L	Start	8.15 (0.57) [19]	8.09 (0.61) [12]	0.56 (0.34-0.79)	7.77	0.50	0.79
	Change	0.01 (0.05) [19]	-0.03 (0.04) [12]	0.79 (0.61-0.96)	-0.03	0.67	0.89
CXCL6	Start	7.32 (0.83) [19]	6.05 (1) [12]	0.82 (0.67-0.97)	5.73	0.50	1.00
	End	8.51 (1.6) [19]	9.98 (1.25) [12]	0.79 (0.62-0.96)	9.13	0.92	0.68
	Change	-0.1 (0.14) [19]	-0.2 (0.11) [12]	0.76 (0.59-0.93)	-0.05	1.00	0.47
CXCL10	Start	10.48 (1.1) [19]	9.8 (1.77) [12]	0.65 (0.42-0.89)	9.31	0.58	0.89
	End	12.46 (1.34) [19]	13.19 (0.57) [12]	0.71 (0.52-0.89)	12.48	0.92	0.58
	Change	-0.16 (0.2) [19]	-0.17 (0.1) [12]	0.64 (0.43-0.86)	-0.15	0.67	0.79
X4E BP1	Start	6.22 (2.24) [19]	4.53 (1.63) [12]	0.75 (0.56-0.93)	4.14	0.50	0.95
	End	7.48 (1.61) [19]	7.84 (0.9) [12]	0.56 (0.35-0.76)	6.13	1.00	0.26
	Change	-0.12 (0.22) [19]	-0.16 (0.08) [12]	0.64 (0.45-0.84)	-0.11	0.83	0.58
	Start	9.5 (0.6) [19]	9.53 (0.42) [12]	0.53 (0.31-0.74)	9.81	0.83	0.37

DNER	End	9.6 (0.58) [19]	10.13 (0.16) [12]	0.82 (0.67-0.97)	9.88	1.00	0.68
	Change	-0.01 (0.04) [19]	-0.03 (0.02) [12]	0.73 (0.55-0.91)	-0.01	0.92	0.58
EN RAGE	Start	4.54 (2.35) [18]	3.5 (1.66) [11]	0.66 (0.44-0.88)	2.67	0.45	0.89
	End	4.48 (1.77) [19]	4.88 (1.79) [12]	0.54 (0.33-0.75)	5.58	0.50	0.68
	Change	-0.02 (0.18) [18]	-0.08 (0.1) [11]	0.68 (0.48-0.88)	0.03	1.00	0.50
CD40	Start	9.78 (1.06) [19]	9.51 (0.96) [12]	0.58 (0.36-0.79)	9.78	0.75	0.53
	End	9.95 (0.67) [19]	10.39 (0.63) [12]	0.66 (0.47-0.86)	10.22	0.67	0.68
	Change	-0.03 (0.1) [19]	-0.04 (0.06) [12]	0.64 (0.43-0.85)	-0.08	0.33	0.95
IFN gamma	Start	5.56 (0.84) [8]	5.91 (0.88) [5]	0.68 (0.35-1)	5.45	0.80	0.62
	End	6.36 (1.18) [15]	5.94 (0.71) [11]	0.6 (0.38-0.82)	6.81	0.91	0.40
FGF 19	Start	5.57 (0.82) [19]	5.7 (0.74) [12]	0.59 (0.38-0.8)	5.15	0.92	0.42
	Change	0.08 (0.06) [19]	0.02 (0.06) [12]	0.74 (0.55-0.92)	0.03	0.75	0.74
LIF	Start	7.86 (2.04) [19]	7.4 (2.54) [12]	0.59 (0.37-0.81)	8.72	0.83	0.42
	End	4.08 (1.4) [19]	5.07 (1.31) [12]	0.7 (0.5-0.89)	4.67	0.67	0.74
	Change	0.26 (0.13) [19]	0.12 (0.15) [12]	0.78 (0.59-0.97)	0.22	0.83	0.68
MCP 2	Start	7.95 (1.5) [19]	7.78 (1.11) [12]	0.5 (0.29-0.72)	9.93	1.00	0.21
	End	8.56 (1.2) [19]	8.97 (0.72) [12]	0.61 (0.4-0.81)	8.25	0.92	0.42
	Change	-0.07 (0.21) [19]	-0.06 (0.07) [12]	0.56 (0.35-0.77)	0.02	1.00	0.32
CASP 8	End	3.85 (0.97) [13]	3.11 (0.49) [7]	0.76 (0.54-0.98)	3.87	1.00	0.54
CCL25	Start	2.92 (0.48) [6]	2.71 (0.46) [5]	0.63 (0.25-1)	2.84	0.80	0.67
CX3CL1	End	2.28 (0.19) [5]	2.41 (0.24) [8]	0.7 (0.39-1)	2.49	0.50	1.00
TNFRSF9	Start	3.96 (0.64) [17]	3.56 (0.41) [6]	0.68 (0.45-0.91)	4.11	1.00	0.41
	End	4.19 (0.59) [19]	4.59 (0.51) [12]	0.69 (0.5-0.89)	3.92	1.00	0.47
	Change	-0.01 (0.04) [17]	-0.04 (0.04) [6]	0.69 (0.4-0.98)	-0.04	0.67	0.82
	Start	8.11 (0.74) [19]	8.16 (0.69) [12]	0.54 (0.32-0.76)	8.04	0.67	0.58

TWEAK	End	8.27 (0.7) [19]	8.75 (0.52) [12]	0.71 (0.53-0.9)	8.30	0.92	0.53
	Change	-0.02 (0.05) [19]	-0.03 (0.04) [12]	0.62 (0.41-0.83)	0.00	0.83	0.47
CCL20	Start	9.01 (2.05) [19]	8.51 (2.07) [12]	0.61 (0.39-0.83)	9.01	0.83	0.58
	End	7.68 (1.93) [18]	6.85 (0.94) [12]	0.61 (0.4-0.82)	7.66	0.92	0.44
	Change	0.11 (0.17) [18]	0.09 (0.11) [12]	0.6 (0.38-0.82)	0.11	0.83	0.56
STAMBP	Start	4.06 (1.95) [19]	3.06 (1.04) [12]	0.65 (0.45-0.85)	3.26	0.83	0.53
	End	2.8 (0.72) [15]	2.9 (0.58) [12]	0.54 (0.31-0.77)	3.05	0.50	0.73
	Change	0.09 (0.1) [15]	0.01 (0.05) [12]	0.77 (0.58-0.95)	0.03	0.75	0.80
ADA	Start	5.53 (1.45) [19]	4.87 (0.92) [12]	0.65 (0.44-0.86)	4.10	0.33	1.00
	End	5.32 (1.04) [19]	5.84 (0.87) [12]	0.63 (0.42-0.83)	5.74	0.58	0.74
	Change	0 (0.08) [19]	-0.05 (0.04) [12]	0.71 (0.53-0.9)	0.00	0.92	0.47
TNFB	End	2.89 (0.64) [17]	2.96 (0.4) [11]	0.59 (0.38-0.81)	2.75	0.73	0.59
CSF 1	Start	9.18 (0.61) [19]	8.71 (0.5) [12]	0.72 (0.53-0.9)	9.14	0.92	0.53
	End	8.39 (0.62) [19]	9.06 (0.35) [12]	0.84 (0.69-0.98)	8.45	1.00	0.68
