# Supplementary material for: Inflammatory Markers as Predictors of Shunt Dependency and Functional Outcome in Patients with Aneurysmal Subarachnoid Hemorrhage
Source: Biomedicines. 2023 Mar 23;11(4):997. doi: 10.3390/biomedicines11040997 (PMC10135965; doi:10.3390/biomedicines11040997)
Supplement: Supplementary file 1 [file biomedicines-11-00997-s001.zip › Rostgaard et al, SAH_SUPPL file2.pdf]

Supplemental file S2, Table S2
Predictors of functional outcome (not reaching lower confidence limit of ≥0.7)

Name	Time	mRS 0-2 - mean (SD) [n]	mRS 3-6 - mean (SD) [n]	AUC (95%CI)	Cut-off	Sensitivity	Specificity
IL8	Start	11.46 (1.66) [12]	11.47 (1.85) [12]	0.51 (0.26-0.75)	13.54	1.00	0.17
IL8	End	11.33 (1.32) [12]	12.94 (0.89) [12]	0.8 (0.6-0.99)	11.32	1.00	0.58
VEGFA	Start	9.86 (0.85) [12]	9.98 (1.07) [12]	0.51 (0.27-0.76)	9.88	0.50	0.75
VEGFA	End	9.76 (0.73) [12]	9.86 (1.17) [12]	0.51 (0.25-0.76)	10.07	0.58	0.75
VEGFA	Change	0.01 (0.05) [12]	0.01 (0.09) [12]	0.55 (0.3-0.79)	0.01	0.58	0.67
CD8A	Start	5.31 (1.16) [12]	5.05 (1.24) [12]	0.57 (0.32-0.82)	4.84	0.58	0.75
CD8A	End	5.51 (0.51) [12]	5.12 (1.4) [12]	0.55 (0.29-0.81)	4.63	0.42	1.00
CD8A	Change	-0.01 (0.06) [12]	0 (0.1) [12]	0.51 (0.25-0.78)	0.01	0.50	0.83
MCP 3	Start	4.74 (1.78) [11]	6.3 (1.87) [9]	0.76 (0.54-0.98)	4.22	1.00	0.55
MCP 3	End	4.54 (1.8) [10]	6.06 (1.65) [12]	0.78 (0.57-0.99)	4.31	1.00	0.60
MCP 3	Change	-0.02 (0.18) [10]	0.02 (0.09) [9]	0.53 (0.25-0.81)	-0.03	0.89	0.40
CDCP1	Start	3.39 (0.41) [12]	3.76 (0.59) [12]	0.72 (0.49-0.94)	3.66	0.67	0.83
CDCP1	End	3.39 (0.49) [12]	3.78 (0.77) [12]	0.71 (0.49-0.93)	3.81	0.58	0.83
CDCP1	Change	-0.01 (0.04) [12]	0 (0.06) [12]	0.52 (0.27-0.77)	0.00	0.58	0.58
CD244	Start	3.56 (0.33) [5]	3.91 (0.88) [6]	0.6 (0.2-1)	4.13	0.50	1.00
OPG	Start	10.26 (1.06) [12]	9.36 (1.21) [12]	0.71 (0.49-0.93)	8.84	0.50	1.00
OPG	End	10.12 (0.93) [12]	9.89 (1.44) [12]	0.53 (0.29-0.78)	10.48	0.75	0.42
OPG	Change	0.03 (0.1) [12]	-0.02 (0.1) [12]	0.59 (0.35-0.83)	-0.05	0.33	0.92
LAP TGF beta 1	Start	4.54 (0.73) [12]	4.11 (0.97) [12]	0.67 (0.44-0.91)	4.01	0.58	0.83
LAP TGF beta 1	End	4.47 (0.57) [12]	4.1 (0.86) [12]	0.68 (0.46-0.91)	4.63	0.83	0.50
LAP TGF beta 1	Change	0 (0.06) [12]	0 (0.08) [12]	0.52 (0.25-0.79)	0.06	0.42	1.00
uPA	Start	7.61 (0.78) [12]	8.02 (0.75) [12]	0.66 (0.43-0.89)	7.03	1.00	0.33
uPA	End	8.75 (1.1) [12]	9.17 (1.36) [12]	0.6 (0.36-0.85)	8.87	0.75	0.58

uPA	Change	-0.09 (0.12) [12]	-0.06 (0.09) [12]	0.55 (0.31-0.79)	-0.06	0.58	0.58
IL6	Start	9.06 (2.08) [12]	8.82 (2.04) [12]	0.54 (0.29-0.79)	8.99	0.67	0.58
IL6	End	7.78 (2.33) [12]	9.08 (1.88) [12]	0.67 (0.44-0.89)	6.32	1.00	0.33
IL6	Change	0.05 (0.15) [12]	-0.01 (0.15) [12]	0.69 (0.46-0.92)	0.05	0.75	0.67
MCP 1	Start	14.71 (0.64) [12]	14.68 (0.43) [12]	0.58 (0.34-0.83)	15.18	1.00	0.25
MCP 1	End	14.39 (0.93) [12]	14.65 (0.78) [12]	0.61 (0.37-0.85)	14.26	0.92	0.33
MCP 1	Change	0.02 (0.03) [12]	0.01 (0.07) [12]	0.65 (0.41-0.88)	0.00	0.58	0.83
CXCL11	Start	4.68 (0.88) [12]	4.88 (1.13) [12]	0.53 (0.29-0.78)	5.53	0.33	0.83
CXCL11	End	6.17 (0.8) [12]	5.76 (1.32) [12]	0.6 (0.37-0.84)	6.69	0.83	0.42
CXCL11	Change	-0.11 (0.12) [12]	-0.05 (0.13) [12]	0.62 (0.38-0.87)	-0.01	0.50	0.92
TRAIL	Start	3.41 (0.96) [10]	3.41 (1.17) [12]	0.5 (0.24-0.76)	2.15	1.00	0.20
TRAIL	End	2.13 (0.31) [10]	2.41 (0.4) [8]	0.79 (0.56-1)	2.14	0.88	0.70
TRAIL	Change	0.06 (0.07) [10]	0.04 (0.06) [8]	0.65 (0.37-0.93)	0.06	0.75	0.70
CXCL9	Start	4.08 (0.81) [12]	4.15 (0.92) [12]	0.53 (0.28-0.77)	3.75	0.75	0.42
CXCL9	End	6.61 (1.4) [12]	6.41 (1.5) [12]	0.55 (0.3-0.79)	7.91	1.00	0.25
CXCL9	Change	-0.17 (0.15) [12]	-0.13 (0.1) [12]	0.56 (0.32-0.81)	-0.10	0.42	0.83
CST5	Start	5.35 (0.2) [12]	5.22 (0.27) [12]	0.65 (0.41-0.88)	5.40	0.83	0.50
CST5	End	5.4 (0.1) [12]	5.38 (0.3) [12]	0.61 (0.36-0.87)	5.50	0.58	0.83
CST5	Change	0 (0.01) [12]	-0.01 (0.02) [12]	0.59 (0.35-0.83)	-0.01	0.50	0.83
OSM	Start	5.79 (1.84) [12]	6.71 (2.07) [12]	0.6 (0.37-0.84)	4.96	0.83	0.42
OSM	End	4.85 (1.5) [12]	5.89 (0.8) [12]	0.75 (0.53-0.97)	4.59	1.00	0.58
OSM	Change	0.03 (0.15) [12]	0.05 (0.12) [12]	0.51 (0.26-0.75)	-0.02	0.42	0.75
CXCL1	Start	9.34 (1.77) [12]	8.9 (1.66) [12]	0.58 (0.33-0.82)	10.96	1.00	0.25
CXCL1	End	10.06 (1.74) [12]	11.32 (1.45) [12]	0.69 (0.47-0.91)	9.74	0.92	0.50
CXCL1	Change	-0.05 (0.08) [12]	-0.13 (0.1) [12]	0.75 (0.54-0.96)	-0.08	0.75	0.83
CCL4	Start	7.71 (2.89) [12]	8.88 (2.11) [12]	0.65 (0.42-0.89)	7.68	0.83	0.50

CCL4	End	6.32 (0.87) [12]	7.53 (1.75) [12]	0.74 (0.53-0.94)	7.10	0.58	0.92
CCL4	Change	0.06 (0.18) [12]	0.07 (0.15) [12]	0.55 (0.3-0.8)	0.05	0.75	0.50
SCF	Start	4.64 (0.81) [12]	4.81 (0.99) [12]	0.53 (0.28-0.79)	3.90	1.00	0.25
SCF	End	4.65 (0.71) [12]	4.82 (1) [12]	0.51 (0.26-0.77)	5.69	0.33	1.00
SCF	Change	0 (0.05) [12]	0 (0.08) [12]	0.51 (0.26-0.77)	-0.01	0.58	0.67
IL18	Start	4.2 (1.49) [12]	4.82 (1.57) [12]	0.6 (0.37-0.84)	3.47	0.83	0.42
IL18	End	4.65 (1.31) [12]	5.53 (0.82) [12]	0.71 (0.48-0.94)	4.42	1.00	0.42
IL18	Change	-0.07 (0.2) [12]	-0.04 (0.1) [12]	0.57 (0.31-0.83)	-0.10	0.50	0.92
TGF alpha	Start	5.54 (1.06) [12]	5.79 (0.92) [12]	0.6 (0.36-0.85)	5.31	0.75	0.58
TGF alpha	End	4.89 (0.51) [12]	4.92 (0.67) [12]	0.54 (0.3-0.79)	4.77	0.67	0.58
TGF alpha	Change	0.04 (0.07) [12]	0.05 (0.07) [12]	0.55 (0.3-0.8)	0.04	0.50	0.83
MCP 4	Start	9.78 (1.78) [12]	10.43 (1.51) [12]	0.58 (0.34-0.83)	8.93	0.92	0.42
MCP 4	End	10.94 (1.48) [12]	11.48 (1.64) [12]	0.62 (0.38-0.85)	11.17	0.75	0.58
MCP 4	Change	-0.09 (0.14) [12]	-0.06 (0.13) [12]	0.53 (0.28-0.79)	0.00	0.42	0.83
CCL11	Start	4.52 (1.1) [11]	4.73 (0.96) [12]	0.55 (0.29-0.81)	3.84	0.92	0.36
CCL11	End	3.19 (0.45) [12]	3.25 (0.4) [12]	0.56 (0.31-0.8)	3.19	0.67	0.58
CCL11	Change	0.07 (0.05) [11]	0.09 (0.06) [12]	0.54 (0.29-0.79)	0.02	0.92	0.27
FGF 5	Start	2.42 (0.52) [10]	2.12 (0.24) [5]	0.64 (0.35-0.93)	2.57	1.00	0.40
FGF 5	End	2.76 (0.57) [11]	2.76 (0.57) [10]	0.51 (0.24-0.78)	2.99	0.40	0.82
FGF 5	Change	-0.03 (0.04) [10]	-0.04 (0.03) [5]	0.58 (0.26-0.9)	-0.01	0.80	0.50
MMP 1	Start	11.53 (2.41) [12]	12.17 (2.26) [12]	0.51 (0.26-0.76)	13.34	0.33	0.92
MMP 1	End	9.51 (1.73) [12]	11.96 (1.82) [12]	0.83 (0.67-1)	10.06	1.00	0.67
MMP 1	Change	0.11 (0.09) [12]	0.01 (0.17) [12]	0.69 (0.46-0.92)	0.12	0.83	0.58
LIF R	Start	3.08 (0.25) [12]	2.9 (0.36) [12]	0.67 (0.44-0.91)	2.88	0.58	0.83
LIF R	End	2.9 (0.48) [12]	2.93 (0.4) [12]	0.58 (0.33-0.83)	2.78	0.83	0.58
LIF R	Change	0.01 (0.03) [12]	0 (0.02) [12]	0.62 (0.39-0.86)	0.00	0.75	0.58

CCL19	Start	9.65 (0.8) [12]	8.96 (1.14) [12]	0.72 (0.5-0.94)	8.70	0.50	1.00
CCL19	End	11.84 (1) [12]	11.12 (1.67) [12]	0.58 (0.34-0.83)	11.21	0.42	0.92
CCL19	Change	-0.15 (0.16) [12]	-0.12 (0.1) [12]	0.54 (0.29-0.79)	-0.08	0.42	0.83
IL 10RB	Start	2.56 (0.49) [11]	2.66 (0.56) [9]	0.54 (0.26-0.81)	2.70	0.44	0.73
IL 10RB	End	2.9 (0.49) [12]	3.16 (0.62) [11]	0.64 (0.39-0.88)	3.05	0.73	0.67
IL 10RB	Change	-0.03 (0.06) [11]	-0.02 (0.05) [9]	0.52 (0.23-0.8)	-0.05	0.33	0.91
IL 18R1	Start	4.61 (0.89) [11]	4.56 (1.31) [12]	0.59 (0.33-0.85)	3.68	0.42	1.00
IL 18R1	End	3.74 (0.6) [11]	3.99 (0.71) [10]	0.63 (0.36-0.9)	3.96	0.60	0.82
IL 18R1	Change	0.03 (0.1) [11]	0.04 (0.09) [10]	0.55 (0.26-0.83)	-0.02	0.40	0.91
PD L1	Start	3.75 (0.32) [9]	3.82 (0.5) [6]	0.52 (0.17-0.87)	3.92	0.50	0.78
PD L1	End	3.78 (0.43) [12]	4 (0.42) [11]	0.64 (0.41-0.88)	3.52	0.91	0.42
PD L1	Change	-0.01 (0.04) [9]	-0.01 (0.05) [6]	0.5 (0.12-0.88)	0.03	0.50	0.89
CXCL5	Start	9.3 (1.85) [12]	9.1 (2.12) [12]	0.53 (0.28-0.78)	7.49	0.33	0.92
CXCL5	Change	0.08 (0.09) [12]	-0.08 (0.16) [12]	0.81 (0.64-0.99)	-0.03	0.58	1.00
TRANCE	Start	1.47 (0.4) [7]	1.97 (0.69) [5]	0.74 (0.4-1)	1.60	0.80	0.71
HGF	Start	7.99 (0.56) [12]	7.96 (0.86) [12]	0.54 (0.28-0.8)	7.42	0.42	1.00
HGF	End	8.17 (0.66) [12]	8.4 (1.2) [12]	0.61 (0.37-0.85)	8.36	0.58	0.67
HGF	Change	-0.02 (0.05) [12]	-0.02 (0.08) [12]	0.62 (0.36-0.87)	-0.03	0.67	0.83
IL 12B	Start	2.16 (0.94) [12]	2.28 (0.96) [12]	0.51 (0.27-0.76)	1.23	1.00	0.17
IL 12B	End	3.3 (1.1) [12]	2.89 (1.14) [12]	0.64 (0.41-0.87)	2.49	0.50	0.83
IL 12B	Change	-0.05 (0.1) [12]	-0.03 (0.1) [12]	0.58 (0.34-0.82)	-0.03	0.50	0.75
MMP 10	Start	6.63 (1.63) [12]	6.47 (1.95) [12]	0.52 (0.27-0.77)	5.42	0.33	0.83
MMP 10	End	5.83 (0.99) [12]	6.83 (1.41) [12]	0.74 (0.51-0.96)	6.91	0.67	0.92
MMP 10	Change	0.08 (0.16) [12]	-0.01 (0.15) [12]	0.63 (0.4-0.87)	-0.04	0.42	0.92
IL10	Start	4.52 (1.26) [10]	4.17 (1.26) [11]	0.58 (0.32-0.84)	3.96	0.55	0.70
IL10	End	3.17 (0.28) [5]	3.17 (0.51) [8]	0.5 (0.16-0.84)	3.48	0.38	1.00

IL10	Change	0.12 (0.09) [5]	0.02 (0.07) [7]	0.77 (0.48-1)	0.04	0.71	0.80
TNF	Start	1.76 (0.98) [11]	1.72 (1.05) [12]	0.52 (0.26-0.77)	1.87	0.75	0.45
TNF	Change	-0.02 (0.1) [11]	-0.09 (0.14) [12]	0.64 (0.41-0.88)	-0.05	0.58	0.73
CCL23	Start	6.2 (0.63) [12]	5.88 (0.97) [12]	0.65 (0.42-0.89)	5.53	0.50	0.92
CCL23	End	7.32 (1.35) [12]	7.82 (1.54) [12]	0.59 (0.35-0.83)	7.48	0.67	0.67
CCL23	Change	-0.07 (0.1) [12]	-0.11 (0.1) [12]	0.58 (0.34-0.82)	-0.06	0.67	0.58
CD5	Start	3.03 (0.99) [12]	3.19 (1.79) [12]	0.53 (0.29-0.78)	2.04	0.33	0.92
CD5	End	3.27 (0.84) [12]	2.81 (0.94) [12]	0.69 (0.47-0.92)	2.22	0.42	1.00
CD5	Change	-0.04 (0.12) [12]	0.03 (0.11) [12]	0.58 (0.33-0.83)	0.03	0.50	0.83
CCL3	Start	7.2 (2.6) [12]	8.23 (2.27) [12]	0.64 (0.4-0.88)	6.80	0.83	0.58
CCL3	End	7.04 (1.24) [12]	9.13 (2.66) [12]	0.79 (0.6-0.98)	7.05	0.92	0.58
CCL3	Change	-0.01 (0.18) [12]	-0.06 (0.15) [12]	0.53 (0.29-0.78)	0.11	1.00	0.25
Flt3L	Start	8.09 (0.45) [12]	8.1 (0.67) [12]	0.55 (0.3-0.8)	7.90	0.58	0.75
Flt3L	End	8.31 (0.57) [12]	8.35 (0.92) [12]	0.6 (0.37-0.84)	8.04	0.83	0.42
Flt3L	Change	-0.02 (0.05) [12]	-0.01 (0.06) [12]	0.56 (0.31-0.81)	-0.04	0.50	0.83
CXCL6	Start	7.18 (0.96) [12]	6.07 (1.02) [12]	0.78 (0.59-0.98)	7.07	0.83	0.67
CXCL6	End	9.28 (1.85) [12]	9.27 (1.77) [12]	0.53 (0.28-0.77)	10.02	0.75	0.50
CXCL6	Change	-0.14 (0.15) [12]	-0.17 (0.14) [12]	0.53 (0.29-0.78)	-0.11	0.83	0.42
CXCL10	Start	10.29 (1.32) [12]	9.6 (1.54) [12]	0.67 (0.45-0.9)	10.29	0.83	0.58
CXCL10	End	12.98 (0.97) [12]	12.69 (1.54) [12]	0.52 (0.28-0.77)	12.32	0.33	0.92
CXCL10	Change	-0.2 (0.24) [12]	-0.17 (0.11) [12]	0.54 (0.29-0.79)	-0.10	0.83	0.42
X4E BP1	Start	5.04 (2.35) [12]	5.64 (2.31) [12]	0.62 (0.39-0.86)	4.16	0.83	0.50
X4E BP1	End	7.29 (1.54) [12]	8.08 (0.7) [12]	0.67 (0.44-0.91)	7.36	0.92	0.58
X4E BP1	Change	-0.17 (0.24) [12]	-0.14 (0.13) [12]	0.57 (0.33-0.81)	-0.14	0.67	0.58
DNER	Start	9.62 (0.37) [12]	9.26 (0.49) [12]	0.72 (0.5-0.93)	9.67	0.83	0.50
DNER	End	9.89 (0.37) [12]	9.84 (0.67) [12]	0.55 (0.3-0.8)	10.09	0.50	0.83

DNER	Change	-0.02 (0.03) [12]	-0.03 (0.03) [12]	0.6 (0.37-0.84)	-0.02	0.75	0.58
EN RAGE	Start	3.43 (1.31) [11]	4.01 (1.92) [11]	0.57 (0.32-0.82)	5.94	0.27	1.00
EN RAGE	End	4.19 (1.57) [12]	5.59 (1.52) [12]	0.74 (0.53-0.94)	3.31	1.00	0.42
EN RAGE	Change	-0.08 (0.17) [11]	-0.08 (0.12) [11]	0.54 (0.28-0.8)	-0.10	0.45	0.73
CD40	Start	9.48 (0.93) [12]	9.65 (0.86) [12]	0.53 (0.29-0.78)	8.71	0.92	0.33
CD40	End	9.97 (0.6) [12]	10.3 (0.8) [12]	0.64 (0.4-0.88)	9.78	0.83	0.50
CD40	Change	-0.05 (0.11) [12]	-0.03 (0.07) [12]	0.53 (0.28-0.77)	-0.05	0.42	0.75
IFN gamma	Start	5.75 (1.04) [5]	5.51 (0.44) [6]	0.57 (0.1-1)	5.59	0.83	0.60
IFN gamma	End	6.51 (1.25) [10]	5.94 (0.85) [10]	0.64 (0.38-0.9)	7.03	1.00	0.40
FGF 19	Start	5.8 (0.68) [12]	5.59 (0.91) [12]	0.57 (0.33-0.81)	5.25	0.50	0.75
FGF 19	End	4.91 (0.28) [12]	4.76 (1.02) [12]	0.62 (0.35-0.89)	4.47	0.50	1.00
FGF 19	Change	0.06 (0.05) [12]	0.05 (0.1) [12]	0.51 (0.26-0.76)	0.00	0.25	1.00
LIF	Start	7.41 (2.09) [12]	7.45 (2.73) [12]	0.51 (0.26-0.76)	9.45	0.33	0.92
LIF	End	4.15 (1.45) [12]	4.84 (1.45) [12]	0.62 (0.39-0.86)	3.81	0.83	0.50
LIF	Change	0.21 (0.11) [12]	0.16 (0.17) [12]	0.59 (0.34-0.85)	0.10	0.50	0.83
MCP 2	Start	7.95 (1.46) [12]	7.96 (1.54) [12]	0.51 (0.26-0.75)	6.83	0.83	0.33
MCP 2	End	8.82 (1.07) [12]	8.74 (1.26) [12]	0.52 (0.27-0.77)	8.93	0.58	0.67
MCP 2	Change	-0.09 (0.23) [12]	-0.04 (0.12) [12]	0.51 (0.25-0.76)	0.01	0.42	0.83
CASP 8	End	3.38 (0.84) [7]	3.62 (0.63) [8]	0.62 (0.3-0.95)	3.18	0.75	0.71
CCL25	Start	2.89 (0.38) [6]	2.75 (0.57) [5]	0.6 (0.19-1)	2.37	0.40	1.00
TNFRSF9	Start	3.77 (0.5) [9]	3.82 (0.54) [7]	0.54 (0.23-0.85)	3.98	0.43	0.78
TNFRSF9	End	4.26 (0.47) [12]	4.49 (0.7) [12]	0.6 (0.36-0.84)	4.95	0.33	1.00
TNFRSF9	Change	-0.02 (0.04) [9]	-0.03 (0.05) [7]	0.51 (0.18-0.84)	0.00	0.43	0.89
TWEAK	Start	8.15 (0.69) [12]	8.06 (0.58) [12]	0.51 (0.27-0.76)	8.98	1.00	0.17
TWEAK	End	8.5 (0.43) [12]	8.59 (0.76) [12]	0.65 (0.4-0.89)	8.34	0.83	0.50
TWEAK	Change	-0.03 (0.06) [12]	-0.03 (0.04) [12]	0.58 (0.34-0.83)	-0.05	0.58	0.75

CCL20	Start	8.1 (2.14) [12]	8.88 (2.27) [12]	0.58 (0.34-0.82)	8.69	0.50	0.75
CCL20	Change	0.13 (0.09) [11]	0.04 (0.17) [12]	0.7 (0.47-0.93)	0.02	0.50	1.00
ST1A1	Start	3.74 (0.75) [6]	4.46 (1.02) [5]	0.83 (0.5-1)	4.56	0.80	1.00
STAMBP	Start	3.43 (1.75) [12]	3.94 (1.62) [12]	0.68 (0.45-0.91)	2.84	0.83	0.58
STAMBP	End	2.77 (0.73) [10]	2.92 (0.61) [12]	0.56 (0.3-0.82)	2.46	0.83	0.40
STAMBP	Change	0.05 (0.1) [10]	0.06 (0.09) [12]	0.57 (0.31-0.82)	-0.01	0.83	0.40
ADA	Start	5.08 (1.41) [12]	5.27 (1.3) [12]	0.6 (0.36-0.84)	4.13	0.92	0.33
ADA	End	5.29 (0.85) [12]	5.89 (0.97) [12]	0.67 (0.44-0.9)	5.96	0.58	0.83
ADA	Change	-0.02 (0.08) [12]	-0.03 (0.07) [12]	0.56 (0.31-0.8)	0.01	0.92	0.33
TNFB	End	3.01 (0.59) [12]	3.03 (0.54) [9]	0.51 (0.25-0.77)	2.55	0.89	0.33
CSF 1	Start	9.03 (0.58) [12]	8.67 (0.48) [12]	0.66 (0.43-0.89)	8.99	0.75	0.58
CSF 1	End	8.72 (0.54) [12]	8.7 (0.7) [12]	0.55 (0.31-0.79)	8.19	0.83	0.33
CSF 1	Change	0.02 (0.04) [12]	0 (0.05) [12]	0.68 (0.45-0.91)	0.00	0.67	0.75
